# Postelimination Cluster of Lymphatic Filariasis, Futuna, 2024

**DOI:** 10.3201/eid3103.241317

**Published:** 2025-03

**Authors:** Clément Couteaux, Thibaut Demaneuf, Laurent Bien, Manuel Munoz, Bernadette Worms, Samuel Chésimar, Gwenael Takala, Atonio Lie, Vincent Jessop, Malia Kalemeli Selemago, Valelia Uhila, Monika Toa, Dominique Euller, Cyrille Goarant

**Affiliations:** Wallis and Futuna Health Agency, Mata’utu, Uvea, Wallis and Futuna (C. Couteaux, L. Bien, M. Munoz, B. Worms, S. Chésimar, G. Takala, A. Lie, V. Jessop, M.K. Selemago, V. Uhila, M. Toa, D. Euller); The Pacific Community, Noumea, New Caledonia (T. Demaneuf, C. Goarant)

**Keywords:** lymphatic filariasis, parasites, neglected tropical disease, vector-borne infections, hotspot transmission, Wallis and Futuna

## Abstract

After detection of 2 clinical lymphatic filariasis (LF) cases in a postelimination context in 2023 on the island of Futuna (Wallis and Futuna archipelago), the Wallis and Futuna Health Agency conducted a LF prevalence survey in Futuna in May 2024. This cross-sectional study, carried out among schoolchildren <18 years of age, identified 5 children with antigenemia, indicating an estimated antigenemia prevalence in Futuna children nearing 2%. The study also confirmed a spatial cluster of cases in the village of Taoa, where the child antigenemia prevalence reached 7.5% (95% CI 2.1%–18.2%), and demonstrated a link between infection and traditional housing. We observed microfilariae in contact cases during secondary investigations. These findings suggest resurgence of LF in a postelimination context, in which the expected child antigenemia prevalence should not exceed 1%. This situation should prompt a new mass drug administration campaign using triple therapy and the reinforcement of epidemiologic and entomologic surveillance.

Lymphatic filariasis (LF) is a vectorborne neglected tropical disease caused by nematode worms. Three worm species can cause the disease, *Brugia malayi*, *B. timori*, and *Wuchereria bancrofti*; the last of those is responsible for LF in Pacific Island countries and territories (PICTs). In 2018, ≈51 million people were infected with LF globally, and by 2021, ≈40 million cases of lymphoedema (i.e., elephantiasis of the lower limbs or hydrocele [scrotal edema] resulting from the blockage of lymph flow in lymphatic vessels) were reported (*1*,*2*).

In 2000, the World Health Organization (WHO) launched the Global Program for the Elimination of Lymphatic Filariasis (GPELF). This program’s strategy is based on mapping LF-endemic areas, reducing filarial transmission through mass drug administration (MDA) of microfilaricides, conducting post-MDA surveillance to document elimination, and implementing postelimination surveillance (*2*,*3*). In 2022, a total of 8 PICTs had reached the “elimination as a public health problem” status, including Wallis and Futuna, and another 8 were in the process of MDA (*4*).

Wallis and Futuna (WF) is an overseas collectivity of France in the Pacific Ocean, 370 km east of Samoa and 800 km west of Fiji (Appendix Figure 1). The territory consists of 2 island groups: Wallis, locally named Uvea (74 km^2^), and Futuna and Alofi (46 km^2^), 230 km southwest of Wallis. Wallis is 1 kingdom with a population of 8,088, whereas Futuna has 2 kingdoms, Alo and Sigave, whose combined populations total 3,063 population, according to a 2023 census (*5*,*6*). The Health Agency (Agence de Santé) is the sole regulator and operator of healthcare services.

Until the 1980s, WF was a hyperendemic area for LF, which was transmitted locally by *Aedes polynesiensis* mosquitoes*.* Microfilaria prevalence rates were 20% in 1958 and 10% by the end of the 1970s (*7*,*8*). Diethylcarbamazine MDA campaigns were implemented throughout the archipelago during 1978–2007; unfortunately, however, no information regarding treatment is available. In 2001, WF joined the Pacific Program for the Elimination of Lymphatic Filariasis, the regional component of the WHO-led GPELF. After a survey reporting a 1% infection prevalence, 6 MDA campaigns with diethylcarbamazine and albendazole were implemented in 2002, 2003, 2004, 2005, 2006, and 2007; reported coverage rates ranged from 53% to 66% (*2*,*9*,*10*). Three transmission assessment surveys (TASs) (a pre-TAS in 2006 and TASs in 2012 and 2016) performed using immunochromatographic card tests reported child prevalence of filarial antigenemia <1% (*10*). In 2018, WHO declared the elimination status of LF (*10*,*11*). Subsequently, no postelimination surveillance was implemented.

In October 2023, a lower limb lymphedema was diagnosed and reported in Wallis in a 70-year-old man. A retrospective investigation of medical records identified another lower limb lymphedema in 2022 in a 34-year-old man and a hydrocele in an 11-year-old child in 2021, both of whom were living on the island of Futuna. All 3 cases were serologically positive for LF (Novalisa IgG ELISA Enzyme Linked Immunoabsorbent Assay; Gold Standard Diagnostics, https://www.goldstandarddiagnostics.cn) and showed marked hypereosinophilia (>1,500 cells/mm^3^ [reference range 40–500 cells/mm^3^]). A search of a laboratory information system identified 109 patients with marked hypereosinophilia during May 15, 2023–May 27, 2024; 57 cases were in Futuna, and 52 were in Wallis, indicating a prevalence of hypereosinophilia 3 times higher in Futuna.

A rapid diagnostic test (RDT) (Bioline Filariasis Test Strip; Abbott, https://www.abbott.com), typically used for TAS, was offered to patients identified on the basis of having hypereosinophilia, leading to the diagnosis of 15 cases of antigenemia (2 in Wallis and 13 in Futuna). In Futuna, 10 of the 13 cases were in patients who lived in the village of Taoa, and none had any clinical signs. Those findings triggered further investigation of the LF situation in Futuna. Our study aimed to assess the prevalence of LF in children <18 years of age in Futuna, specify the spatial distribution of LF cases, and identify factors associated with infection.

## Methods

### Study Design

We conducted a cross-sectional study in all Futuna schools to assess infection status by using an RDT, as recommended by WHO, for postelimination surveillance. We used a non-TAS methodology because WHO does not recommend TAS methodology for postelimination surveillance, given its lack of sensitivity in low-prevalence settings. We assessed possible risk factors for LF by administering a questionnaire and retrieved eosinophil counts from medical records when available. After the school-based study, we screened household contacts of LF-positive children by using secondary surveys.

### Survey Population

We used data from the latest general population census (conducted in 2023). The target population consisted of children <18 years of age living in Futuna, totaling 808 persons, according to a 2023 census. The source population was children <18 years of age attending schools in Futuna, born after the last round of MDA in 2007. The territorial education directorate (Vice Rectorat) provided the list of schoolchildren in Futuna for 2024, which totaled 619 children.

We included children from the source population in the survey if they were enrolled in schools on Futuna Island from 1st grade (6 years of age) to 10th grade (15–16 years of age), if their parents completed the questionnaire and provided written informed consent for testing, and if they attended school on the day of the survey. We excluded children enrolled in special needs classes and those who declined to provide a blood sample. Given the small population size, all 448 eligible children were offered the test.

### Data Collection

Three weeks before the screening, we distributed a survey questionnaire to be self-administered and filled in by parents. We defined LF disease status on the basis of the result of a prospective RDT from a drop of capillary blood taken from the child’s fingertip. We collected information on variables related to exposure to the bites of *Ae. polynesiensis* mosquitoes (the vector of LF in WF), which included information on the type of housing (traditional, permanent house, or hut), the presence of mosquito window screens, and the use of topical mosquito repellents or fumigants. We also asked participants about their perception of mosquito biting intensities. Although other biting insects might contribute to this perception, the limited diversity of mosquito or biting midges species in Futuna mean that *Ae. polynesiensis* mosquitoes probably are the strongest contributor to the perception of biting intensities (*8*,*12*). To our knowledge, no precise evaluation of *Ae. polynesiensis* mosquitoes biting intensity has been conducted in Futuna. However, studies from other Pacific Islands where *Ae. polynesiensis* mosquitoes are present indicate that this species is the cause of a high biting intensity (*13*,*14*). We searched medical records for eosinophil counts from up to 5 years before.

The patient questionnaire included questions regarding a set of sociodemographic variables: identity (surname and first name), date of birth, sex, class, school, and test results with the date of the test. The surveillance team collected a geolocation variable of positive cases by using GPS during secondary surveys of household in which positive cases had been identified.

### Organization of the Survey and Case Definition

We conducted screening in schools in Futuna during May 14–17, 2024, by deploying a team of 4 interviewers who had been formerly trained to perform the RDT. After interviewers verified the child’s identity and parental consent, we provided the child with information about the purpose of the test and asked for oral consent. After obtaining consent, we took a capillary blood sample from the child’s fingertip.

We used the Abbott Bioline Filariasis Test Strip for screening (*15*). We rechecked positive samples by using the same test performed in the laboratory from a venous puncture in the next 2 days to comply with the health regulations of France, which requires confirmation of the RDT in a laboratory setting. We defined a case of LF by a positive filariasis test strip antigenemic test confirmed in the laboratory. We did not evaluate microfilaremia because the technique for doing so was not available in the laboratory.

### Survey of Case-Patient Contacts

After laboratory confirmation of the antigenemic cases detected in the schools, we offered all persons living in the same household as a positive case-patient an RDT on heparinized whole blood, in accordance with WHO recommendations. We also performed a qualitative search for microfilariae by direct search on fresh blood smear.

### Information Flow and Analyses

We handwrote RDT results on each child questionnaire during the survey. We then entered all deidentified data into an Epi Info form (https://www.cdc.gov/epiinfo). We cleaned the data by using Excel (Microsoft, https://www.microsoft.com) and analyzed data by using R version 4.2.1 (The R Project for Statistical Computing, https://www.r-project.org) with R studio 2022.02.3+492 (https://rstudio-desktop.fr.download.it).

We described the dataset using the same terms as in the questionnaire. We combined some variables to create binary choices for the analysis (e.g., inhabitants of tin shacks and fale [a traditional Polynesian house with open sides and a thatched roof] were defined as living in traditional housing). We compared eosinophil counts on the basis of the test result as a quantitative variable, as we did for the child’s age. We calculated prevalence of filarial antigenemia on the basis of the population screened and whether a valid positive or negative RDT result was obtained.

We used Fisher exact tests for qualitative variables and Mann–Whitney–Wilcoxon tests to compare the means of a child’s age and eosinophil concentration. We considered differences with a p value <0.05 to be statistically significant. We used univariate logistic regression to study the association between screening results and risk factors. We excluded data on the presence of mosquito nets from the logistic regression analysis because it was a confounder with the type of habitat. In additionally, the low proportion of cases precluded the interpretation of a multivariate analysis. For the spatial analysis, we considered all known LF cases from Futuna on the basis of the school-based survey and positive contacts of antigenemic children, clinical cases, and antigenemic cases identified from the active eosinophilia-based surveillance, which totaled 21 cases. 

We generated maps by using the Leaflet package in R (*16*). We used the global Moran *I* test, “spdep” R package (*17*) to detect the presence of spatial autocorrelation and to identify a possible cluster of cases. We used SaTScan software and the Kulforff method (*18*) based on a spatial Poisson discrete model to identify clusters by the Monte Carlo method and reported results with their radius, the number of observed and expected cases, the relative risk, and their statistical significance level. In addition, we calculated the barycenter (i.e., the arithmetic mean of the latitudes and longitudes) of the Taoa village cases. We used binomial distribution to estimate the LF prevalence in children. 

### Ethics Considerations

Wallis and Futuna does not have an ethics committee. This survey was presented to and received a favorable recommendation from the Health Agency Medical Committee, acting as an institutional review board, and from WHO. We offered antigenemic case-patients triple therapy with albendazole, diethylcarbamazine, and ivermectin, per WHO recommendations, free of charge.

## Results

### Study and Survey Populations

Of 448 eligible children, 353 returned the questionnaires, completed by their parents, yielding a 79% participation rate. Approximately 83% of the children in the population surveyed took part in the screening, making the screening rate in the eligible population just above 65%. The proportion of the eligible population who had an interpretable test, positive or negative, was 61% (Appendix Figure 2). The mean age of the children participating in the survey was 10.3 years (SD 2.8 years), and the mean eosinophil count was 571 cells/mm^3^ (SD 623 cells/mm^3^). Approximately 54% of participants were girls and 46% boys. Half were attending elementary schools, and half were attending junior high schools or high schools. Almost 60% of the pupils attended a school in the kingdom of Alo. The distribution by class, from 1st to 9th grade, was fairly even (at ≈10% per grade), whereas <5% attended 10th grade. Only 3 villages had >10% representation (Taoa, Ono, and Leava); children from Taoa accounted for ≈17% of the participants. In contrast, the villages of Fiua, Tamana, Tavai, and Vele each had <5% representation in the population sampled (Table 1). The sample proved to be a fair representation of the target population (Appendix Table 1).

**Table 1:** Description of sociodemographic and biologic variables of children <18 years of age screened for lymphatic filariasis, Futuna, May 2024*

Variable	No. patients	Value
Age, y, mean (SD)	353	10.27 (2.79)
Eosinophil concentration, cells/mm^3^, mean (SD)	114	571.00 (623.03)
Sex		
F	191	54.1
M	162	45.9
Elementary school level, 1st–5th grade	178	50.4
Location of school		
Fiua	69	19.6
Kolopelu	109	30.9
Sausau	69	19.5
Sisia	106	30.0
School grade		
10th grade	16	4.5
9th grade	37	10.5
8th grade	39	11.0
7th grade	39	11.1
6th grade	43	12.2
5th grade	30	8.5
4th grade	44	12.5
3rd grade	37	10.5
2nd grade	28	7.9
1st grade	40	11.3
Village of habitation (2023 census population [%])		
Fiua (245 [8.0])	16	4.6
Kolia (237 [7.7])	29	8.2
Leava (302 [9.9])	41	11.6
Malae (155 [5.1])	18	5.1
Nuku (202 [6.6])	28	7.9
Ono (504 [16.5])	46	13.0
Poi (165 [5.4])	19	5.4
Tamana (147 [4.8])	10	2.8
Taoa (443 [14.5])	59	16.7
Tavai (132 [4.3])	13	3.7
Toloke (168 [5.5])	33	9.4
Vaisei (139 [4.5])	13	3.7
Vele (222 [7.2])	28	7.9

*Values are % frequency except as indicated.

The habitation type most reported was permanent house, accounting for 94% of the population studied; 80% of habitations did not report having mosquito nets. More than 30% of participants reported using topical mosquito repellents or pyrethroid-containing mosquito coils. The same proportion rated mosquito biting intensity as “fairly high” or above. Nearly 80% of households raised pigs; 30% of households had >10 pigs. Over 83% of the survey population took part in the infection screening, and nearly 4% were absent from school on the day of the survey. For the 293 children taking the RDT, 20 tests (6.8%) were invalid, 5 (1.7%) were positive, and 268 (91.5%) were negative (Table 2). The high rate of invalid tests was mainly attributable to insufficient sampling, given that the minimum volume of blood for the filariasis test strip test is 75 µL.

**Table 2:** Social and behavioral variables and lymphatic filariasis test results, Futuna, May 2024

Variable and test result	No. patients	Frequency, %
Habitation type		
Tin hut	2	0.6
Closed traditional fale*	6	1.7
Open traditional fale	12	3.4
Permanent house	330	94.3
Mosquito net at habitation		
No	273	77.8
Yes	47	13.4
Partial	31	8.8
Repellent or mosquito coil use		
Never	120	34.2
Sometimes	120	34.2
Often	60	17.1
Very often	15	4.3
Always	36	10.2
Perceived biting density		
Absence of bites	92	26.7
Low	155	44.9
Fairly high	70	20.3
High	19	5.5
Very high	9	2.6
No. pigs at household		
0	74	21.0
1–5	78	22.1
6–10	97	27.5
>10	104	29.4
Lymphatic filariasis rapid diagnostic result		
Negative	268	91.5
Positive	5	1.7
Invalid	20	6.8

*Fale, a traditional Polynesian house with open sides and a thatched roof.

### Estimated Prevalence in the Child Population

**Figure 1:**
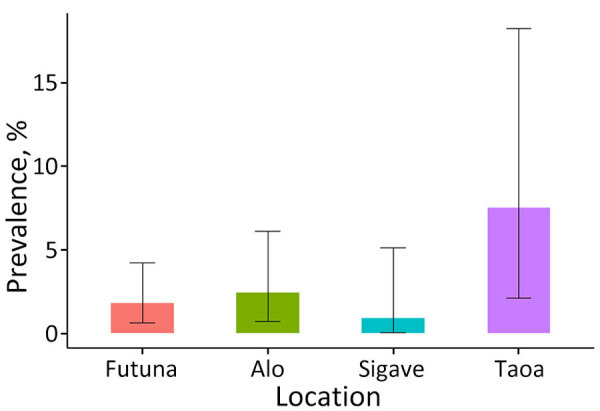
Estimated prevalence of lymphatic filariasis in schoolchildren in Futuna overall, in the kingdoms of Alo and Sigave, and in the village of Taoa, Futuna, May 2024. A total of 283 schoolchildren were screened. Error bars indicate 95% CIs.

Based on the proportion of positive tests among valid readings, the prevalence estimate was 1.8% (95% CI 0.6%–4.2%). The prevalence was 2.4% (95% CI 0.7%–6.1%) in the kingdom of Alo, compared with 0.9% (95% CI 0.0%–5.1%]) in the kingdom of Sigave. Prevalence reached 7.5% (95% CI 2.1%–18.2%) in the village of Taoa (Figure 1).

### Spatial Analysis

**Figure 2:**
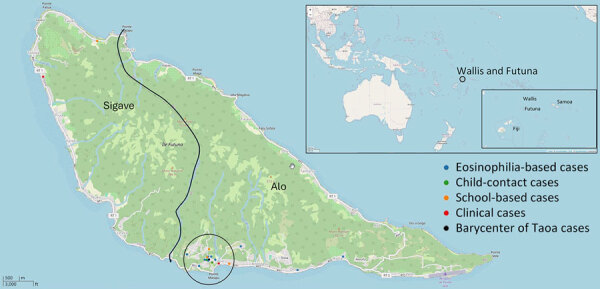
Geolocation of lymphatic filariasis cases detected in Futuna during October 2023–June 2024 and barycenter of Taoa cases. Kingdoms of Alo and Sigave are indicated. Inset maps show location of Wallis and Futuna in South Pacific.

Global Moran index was significantly different from that expected (Moran *I* 0.353; p<0.001), indicating positive spatial autocorrelation and the presence of >1 LF cluster. Clustering analysis with SaTScan revealed a significant cluster in the village of Taoa (relative risk 18.91; p<0.001), which had 16 cases. All cases detected in Taoa were in children who lived 750 m from the barycenter (Figure 2). One case detected during the school survey had already been detected through the recently introduced active surveillance based on hypereosinophilia.

### Factors Associated with Infection

We observed no effect of age on infection status and no difference by school grade (e.g., 1 case in each grade in elementary school except 1st grade and 1 case in 7th grade. One single case was in a child living in the kingdom of Sigave, compared with 4 in the kingdom of Alo. Fisher tests indicated significant differences for village of residence (p = 0.006) and habitation type (p = 0.030). We identified no positive cases in children from households with mosquito nets. In contrast, 15% of LF-negative schoolchildren’s habitations had mosquito nets, a nonsignificant difference. We also observed no significant difference for the use of insect repellents, mosquito coils usage at home, or perceived mosquito biting intensity. All 5 case-patients and 78% of children testing negative reported raising pigs at home (p = 0.59 by Fisher test). Univariable logistic regression models identified associations between testing positive and living in Taoa (odds ratio 17.9 [95% CI 2.0–163.5]; p = 0.003) and living in a traditional habitation (OR 12.0 [95% CI 1.9–77.8]; p = 0.023) (Table 3).

**Table 3:** Univariable logistic regression model results for biologic and risk factors associated with lymphatic filariasis, Futuna, May 2024*

Variable	Univariate OR (95% CI)		p value
Eosinophil count, cells/mm^3^	1.00 (1.00–1.00)		0.092
Age, y	0.87 (0.62–1.22)		0.415
Village of habitation			
Other	Referent		
Taoa	17.90 (2.0–163.5)		0.003
Habitation type			
Permanent house	Referent		
Traditional	12.00 (1.9–77.8)		0.023
Repellent or mosquito coil use			
No	Referent		
Yes	2.10 (0.2–18.6)		0.498
Perceived biting density			
High	Referent		
Low	1.50 (0.2–13.5)		0.715
Pigs at household			
No	Referent		
Yes	20,541,717 (0.0–∞)		0.113

*OR, odds ratio.

### Biologic Factors Associated with Infection

Eosinophil counts were 2.3 times higher in children testing positive compared with children testing negative for LF. However, this difference was not significant in our small dataset (p = 0.2 by Mann–Whitney–Wilcoxon test).

### Contact Investigations

**Figure 3:**
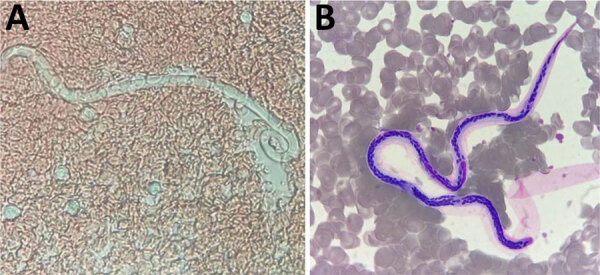
Microphotographs of microfilaria from a sample collected in Futuna, June 2024. A) Fresh blood smear from an antigen-positive child’s close contact; original magnification ×40. B) Smear after May-Grunwald-Giemsa staining; original magnification ×100).

After RDT confirmation of LF in 5 children in the school survey, we offered a screening test to all persons living in the same household as the children with antigenemia. Among these 10 contacts, the father of a 5-year-old child tested positive. A blood smear confirmed microfilaremia in this adult (Figure 3; Video).

**Video:** Microfilariae in fresh blood smear, without hemoconcentration, taken during the screening of contacts of an antigen-positive child, Futuna, June 2024.

## Discussion 

In this postelimination LF prevalence assessment in Futuna, >60% of the eligible population was screened, showing a prevalence of nearly 2% among schoolchildren, twice the expected prevalence in the general population in a postelimination context (*19*). This estimate reached 7.5% in 1 village. Given that the survey population was born after the last MDA round, a much lower prevalence would be expected if transmission had been stopped. This study also identified a link between infection and traditional housing, which posed a significant risk (p = 0.023). The small number of cases limited multivariate analysis and led unprecise results with wide CIs. Although cases were identified through RDTs, microfilaremia was evidenced from fresh blood smears during contact investigations.

The youngest positive case-patient in this study was 7 years of age; earlier eosinophilia-based surveillance had also identified a case in a 6-year-old. Our results are robust and generalizable to the entire child population of Futuna and can be used for public health decision-making.

The 2012 and 2016 TAS surveys screened almost all primary school children, reaching coverage of 90% in 2012 and 88% in 2016, meaning prevalence underestimation is unlikely. In this 2024 study, systematic screening was extended to secondary school children, achieving a 61% screening rate. This reemergence probably has multifactorial causes. The effectiveness of the LF-elimination program depends on administering mass treatments to >65% of the eligible population. In WF, average MDA coverage during 2002–2007 was <58%. Unfortunately, island- or age-specific MDA coverage data is lacking, so whether Futuna had lower coverage or age-specific gaps remain uncertain (*10*). Age-specific MDA heterogeneities have been identified as possible drivers of LF resurgence in Madagascar (*20*).

*Ae. polynesiensis* mosquitoes, LF vectors in WF, are exophilic and reaching high densities in rural and sylvatic environments (*12*). This aggressive exophagous species feeds on humans and animals outdoors (*21*). In 1981, biting intensities nearing 100 bites/hour were reported at dusk in Wallis (*8*); similar findings were reported in Samoa (*13*). The vector’s biology might explain the increased LF risk linked with traditional habitations like the Polynesian fale. The 3.3 male:female sex bias among 21 cases (clinical and antigenemic) since October 2023 probably reflects higher exposure during more male-centered activities, such as agriculture, farming, and sociocultural practices such as traditional kava-drinking ceremonies (Tauasu), during which men remain outdoors from dusk to early night.

No vector-control measures were implemented during 2013–2021 in Futuna, a highly favorable environment for *Ae. polynesiensis* mosquitoes, which are recognized as a good LF vector, even with low microfilaremia (*10*,*22*,*23*). Together with the high vector density and intense biting exposure, vector biology and ecology probably were strong determinants of this reemergence. Xenomonitoring could improve surveillance and guide much-needed vector-control strategies in post-MDA surveillance (*24*).

Furthermore, LF was not a target for postelimination surveillance. The shorter wording “elimination” used without “as a public health problem” probably caused confusion between “elimination” and “eradication.” Consequently, medical staff might not have considered LF as a possible diagnosis in recent years, abandoning passive surveillance altogether.

Active surveillance based on hypereosinophilia initiated in October 2023 used a threshold value of 1,500 cells/mm^3^ to evaluate LF using an RDT. A study in French Polynesia evaluated the predictive performance of eosinophilia for LF infection, establishing a lower threshold value of 500 cells/mm^3^ for optimal sensitivity and specificity (*25*). According to the laboratory information system, 500 patients had eosinophilia above the 500 cell/mm^3^ threshold during June 2023–June 2024, suggesting that many LF cases remain to be identified. Sustaining this surveillance while adopting the lower threshold could increase sensitivity.

This LF surveillance could also be reinforced by integrating a LF RDT during standardized health population surveys aimed at monitoring behavioral risk factors for noncommunicable diseases, including the Global School-Based Health Survey for adolescents and STEPWise for adults conducted on average every 5 years (*26*,*27*). In addition, reinforcing vector-control activities, reducing breeding sites, and promoting individual protection against mosquito bites are essential measures, especially for vulnerable populations living in traditional habitations.

Furthermore, new rounds of MDA could be considered for Futuna, because active surveillance has not shown any resurgence in Wallis. Risk mapping could be refined through xenomonitoring to support this decision. Since 2017, WHO has recommended triple therapy within the GPELF framework (*28*). Adding ivermectin aims to provide longer microfilaricidal activity, further decreasing LF transmission. Ivermectin might also have co-benefits, notably protecting from helminthiases and scabies. However, although triple therapy safety has been reported, its efficacy for subperiodic diurnal LF was not properly evaluated (*29*).

A systematic bibliographic search on PubMed found no reported cluster or postelimination LF resurgence in the 8 PICTs that declared LF elimination since 2016. The reemergence evidenced in Futuna illustrates the importance of postelimination surveillance.

This cross-sectional study estimated LF prevalence in persons <18 years of age born after the last MDA round at ≈2%, much higher than the WHO elimination criterion of <1%. Child prevalence reached 2.5% in the kingdom of Alo and 7.5% in Taoa. Despite a localized cluster in Taoa, 1 case in a child from Sigave with no linkage to Taoa highlighted the need for islandwide interventions. The study also revealed an association between housing type and LF infection, suggesting traditional or precarious housing as a risk factor linked to *Ae. polynesiensis* mosquito biology.

Although hypereosinophilia might be a predictive marker of LF, our study lacked statistical significance, and further studies are required to confirm this predictive value in the specific context of Futuna. The low coverage of MDA administered during 2002–2007, the temporary absence of vector-control measures, and the absence of postelimination surveillance together might explain the resurgence.

These results should have implications on LF elimination programs because they illustrate the critical role of postelimination surveillance and the complexity of its implementation, through the introduction of targeted screening strategies, integrated with larger-scale surveys or xenomonitoring. For countries that have made considerable efforts to achieve elimination, sustaining the elimination status will require implementing efficient postelimination surveillance, particularly in areas where transmission of LF is ensured by *Ae. polynesiensis* mosquitoes. Our study points to the need to step up epidemiologic and entomologic postelimination surveillance and the probable benefits from implementing new rounds of MDA on the island of Futuna to tackle the reemergence of LF. Particular attention should be paid to vulnerable populations who are more affected by this neglected tropical disease, which was no longer considered as a public health problem. This LF reemergence is also a wake-up call for countries that have already reached the status of elimination as a public health problem.

Appendix Additional information about postelimination cluster of lymphatic filariasis, Futuna, 2024.
