# Supplementary figures and images for: Postelimination Cluster of Lymphatic Filariasis, Futuna, 2024
Source: Emerg Infect Dis. 2025 Mar;31(3):488–96. doi: 10.3201/eid3103.241317 (PMC11878298; doi:10.3201/eid3103.241317)

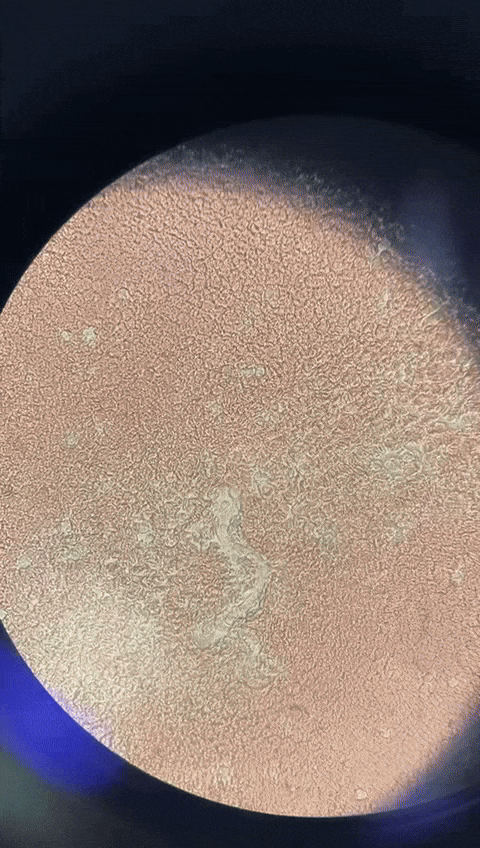

Supplement: Supplementary file 1 [file 24-1317-V.gif]
