# Supplementary material for: Postelimination Cluster of Lymphatic Filariasis, Futuna, 2024
Source: Emerg Infect Dis. 2025 Mar;31(3):488–96. doi: 10.3201/eid3103.241317 (PMC11878298; doi:10.3201/eid3103.241317)
Supplement: Appendix — Additional information about postelimination cluster of lymphatic filariasis, Futuna, 2024. [file 24-1317-Techapp-s1.pdf]

Article DOI: https://doi.org/10.3201/eid3103.241317

EID cannot ensure accessibility for supplementary materials supplied by authors. Readers who have difficulty accessing supplementary content should contact the authors for assistance.

Postelimination Cluster of Lymphatic Filariasis, Futuna, 2024

Appendix

Appendix Table. Comparison of the sample with the target population in Futuna

Socio-demographic	No.	Frequency sample, %	Frequency Futuna population 5–16 y, %
Age class (year)			
5–9	147	41.6	33.6
10–14	186	52.7	47.7
15–16	20	5.7	18.7
Male sex	162	45.9	50.2
Village of habitation			
Fiua	16	4.6	6.6
Kolia	29	8.2	7.8
Leava	41	11.6	10.3
Malae	18	5.1	5.7
Nuku	28	7.9	6.8
Ono	46	13.0	15.4
Poi	19	5.4	4.8
Tamana	10	2.8	3.4
Taoa	59	16.7	14.8
Tavai	13	3.7	5.5
Toloke	33	9.4	6.3
Vaisei	13	3.7	3.1
Vele	28	7.9	9.5

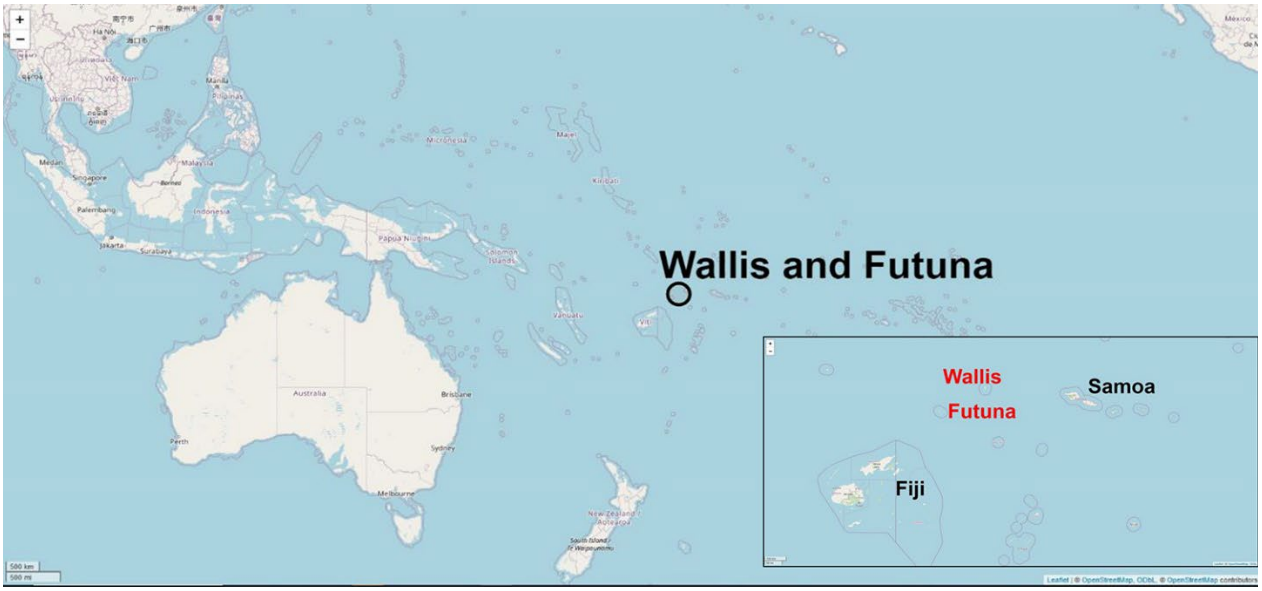

Appendix Figure 1. Geographic location of the Wallis & Futuna territory in the South Pacific.

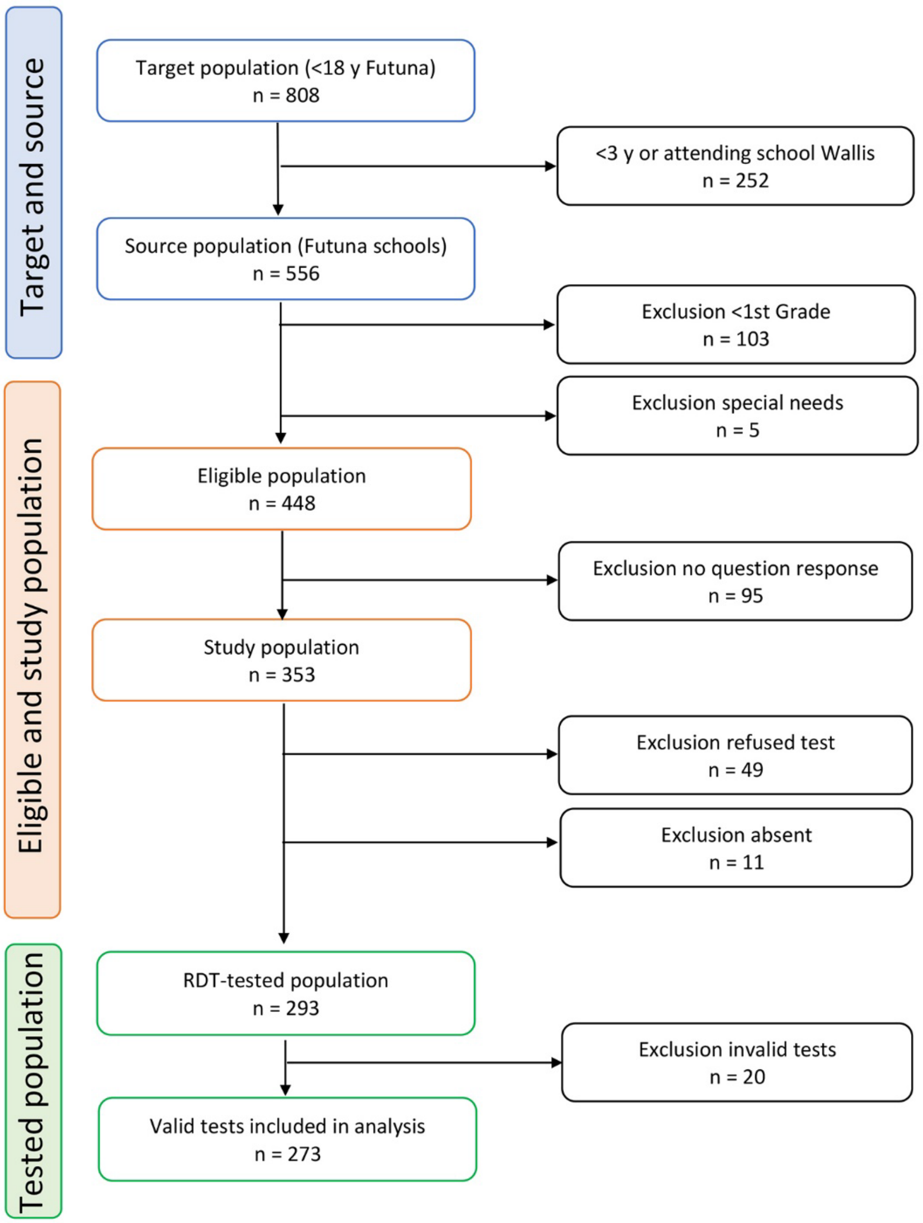

Appendix Figure 2. Flow diagram of the survey population.
